# Supplementing organic-complexed or inorganic Co, Cu, Mn, and Zn to beef cows during gestation: physiological and productive response of cows and their offspring until weaning

**DOI:** 10.1093/jas/skab095

**Published:** 2021-03-24

**Authors:** Kelsey M Harvey, Reinaldo F Cooke, Eduardo A Colombo, Bruna Rett, Osvaldo A de Sousa, Lorin M Harvey, Jason R Russell, Ky G Pohler, Alice P Brandão

**Affiliations:** 1 Department of Animal Science, Texas A&M University, College Station, TX 77845, USA; 2 Prairie Research Unit, Mississippi State University, Prairie, MS 39756, USA; 3 Faculdade de Medicina Veterinária e Zootecnia, Universidade Estadual Paulista, Botucatu, SP 18618-970, Brazil; 4 Pontotoc Ridge-Flatwoods Branch Experiment Station, Mississippi State University, Pontotoc, MS 38863, USA; 5 Zinpro Corporation, Eden Prairie, MN 55344, USA

**Keywords:** beef cows, gestation, offspring, physiology, production, trace minerals

## Abstract

One hundred and ninety non-lactating, pregnant beef cows (three-fourth *Bos taurus* and one-fourth *Bos indicus*; 138 multiparous and 52 primiparous) were assigned to this experiment at 117 ± 2.2 d of gestation (day 0). Cows were ranked by parity, pregnancy type (artificial insemination = 102 and natural service = 88), body weight (**BW**), and body condition score (**BCS**) and assigned to receive a supplement containing: 1) sulfate sources of Cu, Co, Mn, and Zn (**INR**; *n* = 95) or 2) an organic-complexed source of Cu, Mn, Co, and Zn (**AAC**; Availa 4; Zinpro Corporation, Eden Prairie, MN; *n* = 95). The INR and AAC provided the same daily amount of Cu, Co, Mn, and Zn, based on 7 g of the AAC source. From day 0 to calving, cows were maintained in a single pasture and were segregated three times weekly into 1 of the 24 individual feeding pens to receive treatments. Cow BW and BCS were recorded on days −30, 97, upon calving, and at weaning (day 367). Milk production was estimated at 42 ± 0.5 d postpartum via weigh–suckle–weigh (**WSW**) method. Liver biopsies were performed in 30 cows per treatment on days −30, 97, upon calving, and the day after WSW. Calf BW was recorded at birth and weaning. Liver and *longissimus* muscle (**LM**) biopsies were performed in 30 calves per treatment upon calving and 24 h later, the day after WSW, and at weaning. No treatment effects were detected (*P* ≥ 0.49) for cow BCS during gestation, despite AAC cows having greater (*P* = 0.04) BW on day 97. Liver Co concentrations were greater (*P* < 0.01) for AAC compared with INR cows, and liver concentrations of Cu were greater (*P* = 0.02) for INR compared with AAC cows on day 97. Upon calving, INR cows had greater (*P* ≤ 0.01) liver Cu and Zn concentrations compared with AAC cows. No other treatment differences were noted (*P* ≥ 0.17) for cow and calf liver trace mineral concentrations. Cows receiving AAC had greater (*P* = 0.04) hepatic mRNA expression of *metallothionein 1A* at calving, and their calves had greater (*P* = 0.04) hepatic mRNA expression of *superoxide dismutase* at weaning. Milk production did not differ between AAC and INR cows (*P* = 0.70). No treatment effects were detected (*P* ≥ 0.29) for mRNA expression of LM genes associated with adipogenic or muscle development activities in calves at birth and weaning. Calf birth and weaning BW also did not differ (*P* ≥ 0.19) between treatments. In summary, supplementing AAC or INR to beef cows during the last 5 mo of gestation yielded similar cow–calf productive responses until weaning.

## Introduction

Maternal nutrition is a major extrinsic factor programming nutrient partitioning and development of fetal organ systems, leading to long-term effects on offspring production, health, and reproduction (Long et al., 2009, 2010). The majority of research conducted in this area has focused primarily on the energy and protein intake of gestating beef cows (Funston et al., 2010; Bohnert et al., 2013; Wilson et al., 2016), and limited knowledge exists on how trace mineral nutrition during gestation impacts offspring productivity. The fetus is completely dependent on the dam for its supply of trace minerals (Hidiroglou and Knipfel, 1981), which are essential for fetal developmental processes, such as protein synthesis, bone formation, and lipid metabolism (Hostetler et al., 2003). Inadequate maternal intake or transfer of trace minerals to the fetus can result in impaired fetal development and postnatal performance (Weiss et al., 1983).

One strategy to enhance trace mineral status in cattle is to feed organic sources (Spears, 1996). There has been considerable interest in the use of organic trace minerals in ruminant diets due to indications that these may be of greater bioavailability compared with their inorganic counterparts (Brown and Zeringue, 1994). Hostetler et al. (2003) reported that supplementing an organic Cu, Mn, and Zn to gestating sows increased concentrations of these trace elements in fetal tissues and reduced fetal loss by day 30 of gestation compared with sows supplemented with inorganic Cu, Mn, and Zn. Similarly, research from our group reported improved productivity responses in the offspring from beef cows receiving organic trace minerals during late gestation (Marques et al., 2016a). More specifically, supplementing beef cows with organic-complexed Co, Cu, Mn, and Zn enhanced the passage of Zn and Cu to fetal liver, resulting in increased growth rate until weaning and postweaning immunity to bovine respiratory disease.

The results from Marques et al. (2016a) were suggestive of programming effects of organic-complexed trace minerals on postnatal offspring development and health (Funston et al., 2010); however, the physiological mechanisms underlying these outcomes still warranted investigation. Nutritional management of beef cows impacts fetal development throughout the entirety of gestation (Wu et al., 2006), and Marques et al. (2016a) investigated the supplementation for approximately 90 d prior to calving. Hence, supplementing an organic trace mineral source may be even more beneficial to offspring development if offered to beef cows over a greater duration of gestation. To test this theory and expand on the novel results from Marques et al. (2016a), this experiment evaluated the effects of supplementing organic-complexed or sulfate sources of Co, Cu, Mn, and Zn to beef cows during the second and third trimesters of gestation on productive and physiological responses of the cow and their offspring.

## Materials and Methods

This experiment was conducted at the Texas A&M – Beef Cattle Systems (College Station, TX, USA). All animals were cared for in accordance with acceptable practices and experimental protocols reviewed and approved by the Texas A&M – Institute of Animal Care of Use Committee (#2018/0093). This manuscript describes prepartum and postpartum responses of cows as well as offspring responses from birth through weaning. A companion manuscript (Harvey et al., 2021) describes the postweaning responses of the male and female offspring reared, respectively, as feedlot steers or replacement heifers.

### Cow management and dietary treatments

One hundred and ninety non-lactating, pregnant beef cows (average three-fourth *Bos taurus* and one-fourth *Bos indicus*; 138 multiparous and 52 primiparous; initial body weight [**BW**] = 509.3 ± 5.7 kg; age = 4.6 ± 0.2 yr; initial body condition score [**BCS**] = 5.5 ± 0.1 according to Wagner et al., 1988) were assigned to this experiment at 117 ± 2.2 d of gestation (day 0 of the experiment). Cows were pregnant to artificial insemination (**AI**) using semen from a single Brangus sire (*n* = 83) or pregnant to six Brangus bulls according to the breeding management and pregnancy diagnosis described by Oosthuizen et al. (2020).

Prior to the beginning of the experiment (day −30), cows were ranked by pregnancy type (AI or natural service), parity, BW, and BCS and assigned to receive a supplement containing: 1) sulfate sources of Cu, Co, Mn, and Zn (**INR**; custom blend manufactured by Anipro Xtraformance Feeds, Pratt, KS; *n* = 95) or 2) organic-complexed source of Cu, Co, Mn, and Zn (**AAC**; Availa 4; Zinpro Corporation, Eden Prairie, MN; *n* = 95). The AAC trace mineral source was based on a metal:amino acid complex ratio of 1:1 for Zn, Cu, and Mn in addition to cobalt glucoheptonate (Zinpro Corporation). The INR and AAC sources were mixed with dried distillers grain (Table 1) and formulated to provide the same daily amount of energy, protein, macro minerals, and trace minerals, including Cu, Co, Mn, and Zn (based on 7 g/cow daily of Availa 4; as in Marques et al., 2016a). From day 0 of the experiment until calving, cows were maintained in a single pasture dominated by bermudagrass (*Cynodon* spp.). All cows were gathered three times weekly (Tuesdays, Thursdays, and Saturdays) and individually sorted into 1 of the 24 feeding pens (one cow per pen; 6 × 9 m). Cows individually received their assigned supplement treatment (601 or 607 g of INR and AAC per cow each feeding; as-fed basis) and returned to pasture after their treatment was completely consumed. This process was repeated until all cows had been individually sorted into pens and consumed their treatments. From day 93 of the experiment until calving, cows also received daily 12 kg/cow of sorghum-sudangrass hay and 0.76 kg/cow of cubes containing no mineral additive (Table 2).

**Table 1.** Ingredient composition and nutrient profile of supplements containing INR or AAC

Item	INR	AAC
Ingredients, g/d (as-fed basis)		
Dried distillers grains	193	193
Macromineral mix^1^	60.0	60.0
Inorganic trace mix^2^	4.67	0.00
Organic trace mix^3^	0.00	7.00
Nutrient profile^4^ (dry matter [DM] basis)		
DM, %	90.8	90.9
Net energy for maintenance^5^, Mcal/kg	1.58	1.57
Net energy for lactation^5^, Mcal/kg	1.50	1.49
Crude protein, %	26.0	25.7
Ca, %	4.52	4.53
P, %	2.91	2.88
Mg, %	0.64	0.63
K, %	1.13	1.13
Na, %	1.89	1.93
Co, mg/kg	62.0	65.0
Cu, mg/kg	605	600
Fe, mg/kg	1,883	1,931
Mn, mg/kg	983	967
Se, mg/kg	6.44	6.39
Zn, mg/kg	1,791	1,695

^1^Containing (DM basis) 339.7 g/kg CaHPO_4_, 312.7 g/kg CaCO_3_, 197.5 g/kg NaCl, 39.1 g/kg KCl, 18.9 g/kg MgO, 4.0 g/kg Fe_2_O_3_, 2.5 g/kg S, 0.9 g/kg Vit E, 0.8 g/kg Na_2_O_3_Se 3%, 0.7 g/kg Vit A, 0.1 g/kg C_2_H_10_I_2_N_2_, and 0.1 g/kg Vitamin D 400.

^2^Containing (DM basis) 420 g/kg ground corn, 213 g/kg ZnSO_4_, 133 g/kg MnSO_4_, 106 g/kg CuSO_4_, and 8 g/kg CoSO_4_.

^3^Availa 4 (Zinpro Corporation; Eden Prairie, MN), which contained (DM basis) 5.15% Zn from 1:1 Zn and AA complex, 2.86% Mn from 1:1 Mn and amino acid (AA) complex, 1.80% Cu from 1:1 Cu and AA complex, and 0.18% Co from cobalt glucoheptonate.

^4^Values obtained via wet chemistry analysis (Dairy One Forage Laboratory, Ithaca, NY).

^5^Calculated with equations described by the NRC (2000).

**Table 2.** Nutritional profile (dry matter basis) of feedstuffs offered to cows during the experiment^1,2^

Item	Pasture		Supplemental cubes	Sorghum-sudangrass hay
	A	B		
Net energy for maintenance, Mcal/kg	1.14	1.62	2.00	1.00
Net energy for lactation, Mcal/kg	1.18	1.02	1.33	0.44
Crude protein, %	10.7	20.5	48.0	11.8
Neutral detergent fiber, %	57.7	19.6	24.0	61.5
Ca, %	0.98	2.07	0.32	0.76
P, %	0.27	0.32	1.26	0.25
Mg, %	0.29	0.41	0.67	0.31
K, %	1.62	2.72	1.86	4.11
Na, %	0.02	0.05	0.28	0.02
Co, mg/kg	1.61	3.05	1.10	0.69
Cu, mg/kg	8	12	15	11
Fe, mg/kg	3,855	5,120	303	1,566
Mn, mg/kg	106	144	36	54
Se, mg/kg	0.40	0.20	0.19	0.12
Zn, mg/kg	42	35	66	57

^1^Values obtained from a commercial laboratory wet chemistry analysis (Dairy One Forage Laboratory, Ithaca, NY). Total digestible nutrients were calculated according to equations described by Weiss et al. (1992). Net energy for maintenance and lactation were calculated with equations described by the NRC (2000).

^2^Cows were maintained in a single pasture (A) dominated by bermudagrass (*Cynodon* spp.) and were supplemented with 12 kg of sorghum-sudangrass hay and 0.76 kg of cubes daily from day 93 until calving. Upon calving, cow–calf pairs were moved to an adjacent pasture (B) dominated by perennial ryegrass (*Lolium perenne* L.).

After calving, cow–calf pairs were moved to an adjacent pasture dominated by perennial ryegrass (*Lolium perenne* L.) and assigned to the general management of the research herd, which included free-choice inorganic trace mineral supplementation (Producers Special Pasture Mineral; Producers Cooperative Association, College Station, TX; containing 14% Ca, 7% P, 13% NaCl, 5% Mg, 9,900 mg/kg Zn, 2,500 mg/kg Cu, 100 mg/kg I, 4,000 mg/kg Mn, 26 mg/kg Se, 91 IU/g of vitamin A, 10 IU/g of vitamin D3, and 0.05 IU/g of vitamin E). This trace mineral supplement was the same fed to cows prior to the beginning of this experiment. Male calves were castrated using an elastic bander at approximately 30 d of age. Cows were assigned to the same reproductive management (day 214 to 287) and pregnancy diagnosis (day 345 of the experiment) as described by Oosthuizen et al. (2020). Cows were inseminated by the same technician using semen from the same bull and batch and exposed to natural service (six mature bulls) for 60 d. All calves received vaccination against respiratory viruses (Triangle 5; Boehringer Ingelheim Animal Health USA Inc., Duluth, GA) and *Clostridium* (Covexin 8; Merck Animal Health, Omaha, NE) on day 345 of the experiment. Calves were weaned on day 367, when they were revaccinated against respiratory viruses (Titanium 5; Elanco Animal Health, Greenfield, IN) and *Clostridium* (Covexin 8; Merck Animal Health), and received an anthelmintic (Dectomax; Zoetis, Florham Park, NJ).

### Sampling

Samples of all ingredients fed to gestating cows were collected monthly during the experiment, pooled across months, and analyzed for nutrient content by a commercial laboratory (Dairy One Forage Laboratory, Ithaca, NY). The nutritional profile of all ingredients is described in Tables 1 and 2. Individual BW and BCS (Wagner et al., 1988) were recorded from all cows prior to the beginning of the experiment (day −30) and during late gestation (day 97). Liver samples were collected via needle biopsy (TruCut biopsy needle; CareFusion Corporation, San Diego, CA) from 30 cows on days −30 and 97, whereas cows were randomly chosen from each treatment on each sampling day. The same biopsy and selection procedure was adopted for the liver and *longissimus* muscle (**LM**) samplings throughout the experimental period (Marques et al., 2016a; Schubach et al., 2019).

After calving, cow BW and BCS were recorded from all cows, while another subset of 30 cows were randomly selected from each treatment for colostrum sampling via hand milking (100 mL) and liver biopsy. Birth BW and sex were recorded from all calves, and calves from cows selected for colostrum sampling were assigned to liver and LM biopsies. Cows and calves were sampled immediately after calving was identified and completed, except for cows that calved at night whose calves were sampled the next morning within 8 h of calving. Another liver biopsy was collected from the same set of calves 24 h after birth. When feasible, the expelled placenta was retrieved and immediately rinsed with nanopure water for 5 min, with 32 placentas retrieved (16 placentas/treatment). All placentas were expelled within 12 h after calving and not considered retained fetal membranes (Takagi et al., 2002). The five largest cotyledons were dissected from each placenta using curved scissors, given that the largest cotyledons are expected to be the most active regarding the nutrient transfer from the dam to the fetus (Senger, 2003).

Milk production was estimated from all cows, at 42 ± 0.5 d postpartum, via the weigh–suckle–weigh (**WSW**) method (Aguiar et al., 2015). More specifically, calves were separated from their dams for 8 h, weighed, allowed suckling for 30 min, and were weighed again. Milk yield was calculated as the difference in pre- and post-suckling calf BW and was adjusted to 24 h by multiplying by a factor of three. The day following WSW, another subset of 30 cow-calf pairs were randomly selected from each treatment for sampling of fresh milk via hand milking (100 mL) as well as liver biopsy from cows and calves. At weaning (day 367 of the experiment), cow BW and BCS were recorded at weaning, and another subset of 30 calves per treatment were randomly selected for liver and LM biopsy. Calf BW was recorded on days 367 and 368, and the average was considered as calf weaning BW.

### Laboratorial analyses

All feed samples were analyzed by wet chemistry procedures for concentrations of crude protein (method 984.13; AOAC, 2006), acid detergent fiber (method 973.18 modified for use in an Ankom 200 fiber analyzer, Ankom Technology Corp., Fairport, NY: AOAC, 2006), and neutral detergent fiber (Van Soest et al., 1991; modified for use in an Ankom 200 fiber analyzer, Ankom Technology Corp.), macro- and trace minerals using inductively coupled plasma emission spectroscopy (Sirois et al., 1991), and Se according to method 996.16 of the AOAC (2006). Calculations for total digestible nutrients used the equation proposed by Weiss et al. (1992), and calculations for net energy for maintenance and lactation used the equations proposed by NRC (2000).

Liver, LM, cotyledon, colostrum, and milk samples were placed immediately on ice after collected and processed for storage within 8 h. Liver and LM samples were stored in duplicates at −80 °C, with or without 1 mL of RNA stabilization solution (RNAlater, Ambion, Inc., Austin, TX). Liver, cotyledon, colostrum, and milk samples were analyzed via inductively coupled plasma mass spectrometry for concentrations of Co, Cu, Mn, and Zn by the Michigan State University Diagnostic Center for Population and Animal Health (East Lansing, MI) according to Braselton et al. (1997). Total RNA was extracted from liver and LM samples stored in RNA stabilization solution using the TRIzol Plus RNA Purification Kit (Invitrogen, Carlsbad, CA). Quantity and quality of isolated RNA were assessed via UV absorbance (NanoDrop Lite; Thermo Fisher Scientific, Wilmington, DE) at 260 nm and 260/280 nm ratio, respectively (Fleige and Pfaffl, 2006). Reverse transcription of extracted RNA and real-time reverse-transcription polymerase chain reaction (**PCR**) using gene-specific primers (20 pM each; Table 3) were completed as described by Rodrigues et al. (2015). Responses from the genes of interest were quantified based on the threshold cycle (**CT**), the number of PCR cycles required for target amplification to reach a predetermined threshold. A portion of the amplified products was purified with the QIAquick PCR purification kit (Qiagen Inc., Valencia, CA) and sequenced at the Texas A&M AgriLife Genomics and Bioinformatics Service to verify the specificity of amplification. All amplified products represented only the genes of interest. The CT responses from the liver genes of interest were normalized to the geometrical mean of CT values of *ribosomal protein L12* and *cyclophilin*, whereas the CT responses from the muscle genes of interest were normalized to the geometrical mean of CT values of *ribosomal protein S9* and *β-actin* (Vandesompele et al., 2002). The coefficient of variation for the geometrical mean of reference genes across all liver and LM samples was 3.0% and 2.6%, respectively. Results are expressed as relative fold change (2^−ΔΔCT^) as described by Ocón-Grove et al. (2008).

**Table 3.** Primer sequences, accession number, and reference for gene transcripts analyzed by real-time reverse transcription PCR

Target	Primer sequence	Accession no.	Source
Liver samples			
*CUT*			
Forward	GGGTACCTCTGCATTGCTGT	NM_001100381	Han et al. (2009)
Reverse	ATGGCAATGCTCTGTGATGT		
*MT*			
Forward	ATCCGACCAGTGGATCTGTTTGCC	NM_001040492.2	Gessner et al. (2013)
Reverse	AGACACAGCCCTGGGCACACT		
*SOD*			
Forward	TGTTGCCATCGTGGATATTG	NM_174615	Gessner et al. (2013)
Reverse	CAGCGTTGCCAGTCTTTGTA		
*Ribosomal protein L12*			
Forward	CACCAGCCGCCTCCACCATG	NM_205797.1	Gessner et al. (2013)
Reverse	CGACTTCCCCACCGGTGCAC		
*Cyclophilin*			
Forward	GGTACTGGTGGCAAGTCCAT	NM_178320.2	Moriel et al. (2014)
Reverse	GCCATCCAACCACTCAGTCT		
LM samples			
*FABP4*			
Forward	AAACTTAGATGAAGGTGCTCTGG	AJ4160220	Li et al. (2011)
Reverse	CATAAACTCTGGTGGCAGTGA		
*Myogenin*			
Forward	GAGAAGCGCAGACTCAAGAAGGTGAATGA	AF09174	Muroya et al. (2002)
Reverse	TCTGTAGGGTCCGCTGGGAGCAGATGATC		
*PAX7*			
Forward	GGGCTCAGATGTGGAGTCAG	XM_616352.6	Moriel et al. (2014)
Reverse	GCTCCTCTCGGGTGTAGATG		
*PPAR-γ*			
Forward	GCATTTCCACTCCGCACTAT	AY137204	Li et al. (2011)
Reverse	GGGATACAGGCTCCACTTTG		
*Β-actin*			
Forward	AGCAAGCAGGAGTACGATGAGT	NM_173979	Bong et al. (2012)
Reverse	ATCCAACCGACTGCTGTCA		
*Ribosomal protein L12*			
Forward	CCTCGACCAAGAGCTGAAG	AF479289	Jeong et al. (2012)
Reverse	CCTCCAGACCTCACGTTTGTTC		

### Statistical analysis

All cow and calf variables were analyzed with cow as the experimental unit and cow(treatment × parity) as the random variable. Quantitative data were analyzed using the MIXED procedure of SAS (SAS Inst. Inc., Cary, NC), and binary data were analyzed using the GLIMMIX procedure of SAS (SAS Inst. Inc.). All data collected beginning at calving were analyzed using gestation days receiving treatment as an independent covariate. Model statements for cow-related responses included the effects of treatment, parity, day for repeated measures, and all the resultant interactions. Model statements for calf-related responses and placental cotyledons analysis included the effects of treatment, parity, calf sex, day for repeated measures, and all resultant interactions. For repeated measures, the subject for the repeated statement was cow(treatment × parity) and the covariance structure utilized was autoregressive, which provided the best fit according to the lowest Akaike information criterion. Results are reported as least square means (covariately adjusted for post-calving responses) and separated using least square difference. Significance was set at *P* ≤ 0.05, and tendencies were determined if *P* > 0.05 and ≤ 0.10. Results are reported according to main treatment effects if higher-order interactions containing treatments were nonsignificant or according to highest-order interaction detected.

## Results and Discussion

Total feed intake of cows during gestation was not monitored individually as in Marques et al. (2016a) to calculate the daily consumption of Zn, Cu, Mn, and Co. Nonetheless, supplement treatments provided to each cow on a daily basis, approximately 400 mg of Zn, 140 mg of Cu, 230 mg of Mn, and 1.50 mg of Co (Table 1). Based on the BW of cows during gestation (~450 kg), composition of treatments and feedstuffs (Tables 1 and 2), and estimated daily feed intake of 9.0 kg/cow (dry matter basis; NASEM, 2016), diets fed during gestation contained at least 85 ppm of Zinc, 26.6 ppm of Cu, 129 ppm of Mn, and 3.28 ppm of Co. As in Marques et al. (2016a), requirements for Cu, Mn, and Zn were exceeded by more than 200%, whereas the requirement for Co was exceeded by over 2,000% for both INR and AAC treatments (NASEM, 2016). A non-supplemented group was not included in this experiment to conform with current industry practices as the majority of U.S. cow–calf herds receive supplementation (USDA-APHIS, 2010), whereas basal dietary ingredients already provided Co, Cu, Mn, and Zn near or above adequate levels (Table 2; NASEM, 2016). Therefore, this experiment was designed to compare supplemental INR or AAC to gestating beef cows and not to investigate trace mineral deficiency or differences in trace mineral intake between experimental treatments.

### Cow responses

Cow age, BW, and BCS on day −30 were similar (*P* ≥ 0.35) between treatments as designed (Table 4). Length of treatment administration also did not differ (*P* = 0.91) between INR and AAC cows (Table 4), and cows received treatments during the second and third trimesters of gestation. Cows receiving AAC had greater (*P* = 0.04; Table 4) BW at late gestation (day 97) compared with INR cows, although this difference was insufficient to impact concurrent BCS (*P* = 0.93; Table 4). No differences were detected (*P* ≥ 0.31) for cow calving BW and BCS between treatments (Table 4). These outcomes were expected given that AAC and INR cows were maintained in a single group under equivalent nutritional management until calving. Others have also reported that Cu, Co, Mn, and Zn supplementation as organic or inorganic sources resulted in similar cow BCS change (Stanton et al., 2000; Ahola et al., 2004; Marques et al., 2016a). It should be noted that both AAC and INR cows lost BCS during gestation (day effect; *P* < 0.01) due to inclement weather and excessive pasture flooding. Cows should gain BCS during gestation to optimize offspring productivity (Bohnert et al., 2013; Marques et al., 2016b). This BCS loss may have impacted the outcomes of this experiment but equivalently across treatments given the lack of BCS differences (*P* ≥ 0.49) during gestation between AAC and INR cows (Table 4).

**Table 4.** Performance responses of beef cows that received diets containing supplemental INR (*n* = 95) or AAC (*n* = 95) during gestation^1,2^

Item	INR	AAC	SEM	*P-*value
Cow age, yr	4.47	4.60	0.25	0.71
Days receiving treatments, d	167	166	3	0.91
BW, kg				
Initial	485	496	8	0.35
Late gestation	428	443	5	0.04
Calving	419	437	12	0.31
Weaning	463	469	6	0.48
BCS				
Initial	5.44	5.51	0.07	0.49
Late gestation	4.14	4.15	0.07	0.93
Calving	4.50	4.54	0.07	0.73
Weaning	5.26	5.02	0.06	<0.01

^1^INR and AAC cows received the same amount of supplemental Co, Cu, Mn, and Zn from sulfate sources or Availa 4 (Zinpro Corporation, Eden Prairie, MN). Cows were assigned to the experiment at 117 ± 2 d of gestation (day 0).

^2^BW and BCS (Wagner et al., 1988) were recorded before the beginning of the experiment (day −30; initial), on day 97 (late gestation), upon calving, and at weaning (day 367).

No treatment differences were detected (*P* ≥ 0.39) for initial (day −30) liver Cu, Co, Mn, and Zn concentrations (Table 5), indicating similar trace mineral status between AAC and INR cows prior to treatment administration. In samples collected during late gestation (day 97), the liver Co concentrations were greater (*P* < 0.01) for AAC compared with INR cows, and the liver concentrations of Cu were greater (*P* = 0.02) for INR compared with AAC cows. No treatment differences were detected (*P* ≥ 0.27) for liver Mn or Zn concentrations on day 97 (Table 5). Marques et al. (2016a) also reported greater liver Co, less liver Cu, and similar liver Zn and Mn between cows supplemented with sulfate or organic-complexed sources in samples collected during late gestation. Upon calving, INR cows had greater (*P* ≤ 0.01) liver Cu and Zn concentrations compared with AAC cows, and no treatment differences were detected (*P* ≥ 0.17) for liver Mn or Co concentrations (Table 5). Collectively, treatment effects on liver trace mineral status do not corroborate that organic trace mineral sources have increased absorption and retention compared with sulfate minerals (Spears, 1996; Hostetler et al., 2003). Hepatic Mn concentrations are not influenced by dietary Mn intake in ruminants (Underwood and Suttle, 1999; Ahola et al., 2004), and Marques et al. (2016a) reported that Mn supplementation as AAC or INR did not increase liver concentrations of this trace mineral in late-gestating cows. In turn, the effects of supplementing organic Zn, Cu, and Co on liver mineral status in beef cows have been variable (Stanton et al., 2000; Ahola et al., 2004; Arthington and Swenson, 2004). Liver concentrations are often used as the standard for assessing mineral status in livestock but should not be used as an absolute indicator of trace mineral status (McDowell, 2003). Nonetheless, liver Cu concentration in AAC cows was only marginal upon calving (Kincaid, 2000) and decreased by 63% compared with the levels observed in INR cows. Inter-animal variation may have contributed to these unexpected outcomes as animals were chosen at random within each treatment group on each biopsy date; however, attempts to identify definitive biological or experimental explanations would ultimately be highly speculatory. Both AAC and INR cows consumed similar amounts of supplemental Cu during the experiment, and marginal Cu levels noted in AAC cows should not be associated with inadequate intake of this trace mineral during gestation.

**Table 5.** Liver concentrations of Co, Cu, Mn, and Zn in beef cows that received diets containing supplemental INR (*n* = 95) or AAC (*n* = 95) during gestation^1,2^

Item	INR	AAC	SEM	*P-*value
Co, mg/kg				
Initial	0.165	0.155	0.009	0.44
Late gestation	0.586	0.678	0.024	0.01
Calving	0.584	0.550	0.114	0.83
Early lactation	0.140	0.154	0.009	0.31
Cu, mg/kg				
Initial	74.0	66.9	11.0	0.65
Late gestation	125	81.9	12.9	0.02
Calving	118	42.9	9.9	<0.01
Early lactation	136	154	15	0.39
Mn, mg/kg				
Initial	8.94	8.58	0.29	0.39
Late gestation	10.5	10.0	0.3	0.30
Calving	12.0	10.6	0.7	0.17
Early lactation	8.40	7.48	0.47	0.18
Zn, mg/kg				
Initial	148	139	7	0.40
Late gestation	307	341	21	0.27
Calving	173	129	11	0.01
Early lactation	139	155	9	0.21

^1^INR and AAC cows received the same amount of supplemental Co, Cu, Mn, and Zn from sulfate sources or Availa 4 (Zinpro Corporation, Eden Prairie, MN). Cows were assigned to the experiment at 117 ± 2 d of gestation (day 0).

^2^Liver samples were collected before the beginning of the experiment (day −30; initial), on day 97 (late gestation), upon calving when cows were 43 ± 0.5 d postpartum (early lactation), and at weaning (day 367) via needle biopsy (Arthington and Corah, 1995). Concentrations of Co, Cu, Mn, and Zn were determined by the Michigan State University Diagnostic Center for Population and Animal Health (East Lansing, MI; Braselton et al., 1997). Samples were collected from 30 cows of each treatment randomly selected on each sampling day.

No treatment differences were detected (*P* ≥ 0.18; Table 6) for liver mRNA expression of *Cu-transporter protein* (***CUT***), *metallothionein 1A* (***MT***), and *copper-zinc*-*superoxide dismutase 1* (***SOD***) prior to treatment administration (day −30) or during late gestation (day 97). At calving, *MT* expression was greater in AAC vs. INR cows (*P* = 0.04), but no treatment differences were detected for mRNA expression of *CUT* or *SOD* (*P* ≥ 0.19; Table 6). These genes are associated with Cu and Zn metabolism in the liver and provide additional insight into Zn and Cu status of AAC and INR cows during the experiment. The *CUT* is associated with Cu transport into hepatic cells and distribution of Cu into cellular organelles (Prohaska and Gybina, 2004; Han et al., 2009), and its mRNA expression partially modulated by liver Cu (Bauerly et al., 2005; Fry et al., 2013). The *MT* is a superfamily of intracellular metal-binding proteins abundantly found in the liver, with Zn being the primary inductor of its synthesis in hepatic cells (Coyle et al., 2002). Hepatic *MT* expression may be an indicator of Zn bioavailability, given that local Zn levels dictate *MT* mRNA levels (Wang et al., 2012). The SOD is an enzyme that uses Cu or Zn as cofactors and protects cells from oxidative damage by eliminating superoxide anion radicals (Sturtz et al., 2001; Miao and St. Clair, 2009). Hepatic *SOD* mRNA expression was negatively associated with Cu intake and the overall status in cattle (Hansen et al., 2009). In this experiment, AAC cows had greater mRNA expression of *MT* and similar mRNA expression of *CUT* and* SOD* compared with INR cows at calving, despite having less liver Cu and Zn concentration. These outcomes suggest that Cu and Zn status between AAC and INR cows may have not differed during gestation and upon calving as denoted by liver concentrations of these trace minerals.

**Table 6.** Expression of liver genes in beef cows that received diets containing supplemental INR (*n* = 95) or AAC (*n* = 95) during gestation^1,2^

Item	INR	AAC	SEM	*P-*value
*CUT*				
Initial (day −30)	1.86	1.43	0.21	0.18
Pre-calving (day 97)	1.67	1.70	0.08	0.82
Calving	1.95	1.75	0.11	0.19
*MT*				
Initial (day −30)	3.39	2.38	0.73	0.36
Pre-calving (day 97)	26.1	22.8	3.8	0.54
Calving	36.4	65.6	11.6	0.04
*SOD*				
Initial (day −30)	2.29	2.76	0.27	0.24
Pre-calving (day 97)	1.99	2.11	0.13	0.51
Calving	2.02	2.04	0.13	0.90

^1^INR and AAC cows received the same amount of supplemental Co, Cu, Mn, and Zn from sulfate sources or Availa 4 (Zinpro Corporation, Eden Prairie, MN). Cows were assigned to the experiment at 117 ± 2 d of gestation (day 0).

^2^Liver samples were collected via needle biopsy (Arthington and Corah, 1995) before the beginning of the experiment (day −30; initial), on day 97 (late gestation), and upon calving. Values are expressed as relative fold change compared within CT of reference genes analyzed within the same sample (Ocón-Grove et al., 2008). Samples were collected from 30 cows of each treatment randomly selected on each sampling day.

Both AAC and INR cows were in early lactation and with similar (*P* = 0.69) days postpartum at the time of WSW (Table 7). No treatment differences were detected (*P* ≥ 0.19) for milk production as well as milk Co, Cu, Zn, and Mn concentrations (Table 7). Liver concentrations of these trace minerals the day after WSW also did not differ (*P* ≥ 0.18) between treatments (Table 5). Pregnancy rates to AI or overall (AI + bull breeding) did not differ (*P* ≥ 0.41) between INR and AAC cows (46.2% and 40.3% to AI, SEM = 6.0; 68.6% and 62.8% overall, SEM = 5.0; respectively). At weaning, no treatment differences were detected for cow BW (*P* = 0.31), while BCS was greater (*P* < 0.01) in INR compared with AAC cows (Table 4). Collectively, the lack of major treatment differences in measures obtained from WSW until weaning can be attributed to the similar nutritional management that AAC and INR cows received after calving, including inorganic trace mineral supplementation. Treatment differences in BCS at weaning do not follow this rationale, although both INR and AAC cows were within the threshold for adequate BCS in beef females (Richards et al., 1986). Nonetheless, similar milk production and profile between AAC and INR cows indicate that any potential treatment effects on offspring responses were not caused by altered milk yield during early lactation. Hence, supplementing AAC or INR to gestating beef cows did not impact their post-calving lactation and reproductive performance, corroborating the results from previous research in this area (Stanton et al., 2000; Muehlenbein et al., 2001; Marques et al., 2016a).

**Table 7.** Lactation responses of beef cows that received diets containing supplemental INR (*n* = 95) or AAC (*n* = 95) during gestation^1^

Item	INR	AAC	SEM	*P-*value
Days in milk	42.6	42.2	0.7	0.69
Milk yield, kg/d	6.62	6.98	0.70	0.70
Milk mineral profile^2^				
Co, mg/kg				
Colostrum	0.0052	0.0055	0.0003	0.50
Milk	0.00030	0.00035	0.00004	0.40
Cu, mg/kg				
Colostrum	0.985	0.997	0.014	0.58
Milk	0.049	0.062	0.007	0.19
Mn, mg/kg				
Colostrum	0.061	0.055	0.007	0.53
Milk	0.014	0.016	0.002	0.42
Zn, mg/kg				
Colostrum	15.3	13.7	1.6	0.48
Milk	4.21	4.26	0.30	0.89

^1^INR and AAC cows received the same amount of supplemental Co, Cu, Mn, and Zn from sulfate sources or Availa 4 (Zinpro Corporation, Eden Prairie, MN). Cows were assigned to the experiment at 117 ± 2 d of gestation (day 0). Colostrum samples were collected manually upon calving. Milk production was estimated via the WSW method (Aguiar et al., 2015), and milk samples were manually collected the day after WSW. Colostrum and milk samples were collected from 30 cows of each treatment randomly selected on each sampling day.

^2^Concentrations of Co, Cu, Mn, and Zn were determined by the Michigan State University Diagnostic Center for Population and Animal Health (East Lansing, MI; Braselton et al., 1997).

### Calf birth to weaning responses

No treatment differences were detected (*P* ≥ 0.51; Table 8) for calving rate and calf birth BW. No treatment differences were also detected (*P* ≥ 0.21) for the proportion of calves born to AI or proportion of male calves born (Table 8), whereas sex and genetic merit impact offspring developmental responses (Koger and Knox, 1945). Previous research also reported similar calf birth BW when supplementing gestating beef cows with inorganic or organic sources of trace minerals (Stanton et al., 2000; Sprinkle et al., 2006; Marques et al., 2016a). Hence, fetal growth was likely similar between AAC and INR cows despite differences detected for liver concentrations of Co, Zn, and Cu during gestation.

**Table 8.** Calving and weaning outcomes from beef cows that received diets containing supplemental INR (*n* = 95) or AAC (*n* = 95) during gestation^1^

Item	INR	AAC	SEM	*P-*value
Calving results				
Calving rate, %	97.8	99.0	1.3	0.51
% of male calves born	60.7	49.0	7.2	0.21
% of calves born from AI	52.8	54.1	5.2	0.86
Calf birth BW, kg	30.5	30.8	0.5	0.59
Adjusted calf birth BW^2^, kg	32.5	32.9	0.5	0.60
Weaning results				
Weaning rate, %	91.2	89.9	3.0	0.76
% of male calves weaned	60.0	49.5	7.5	0.26
% of weaned calves from AI	50.6	55.0	5.4	0.56
Calf weaning age, d	199	200	3	0.79
Calf weaning BW, kg	183	178	3	0.19
205-d adjusted weaning BW^2^, kg	192	187	3	0.18

^1^INR and AAC cows received the same amount of supplemental Co, Cu, Mn, and Zn from sulfate sources or Availa 4 (Zinpro Corporation, Eden Prairie, MN). Cows were assigned to the experiment at 117 ± 2 d of gestation (day 0). Calves were weaned on day 367 of the experiment.

^2^Calculated according to Beef Improvement Federation (2010).

No treatment differences were detected (*P* ≥ 0.61) for Co, Cu, Mn, or Zn concentrations in the placental cotyledons (Table 9). No treatment differences were also detected (*P* ≥ 0.51) for liver Co, Cu, Mn, or Zn concentrations in calves at birth or 24 h after birth (Table 9) or concentrations of these trace minerals in the colostrum (*P* ≥ 0.48; Table 7). The fetus relies completely on the dam for proper supply of trace minerals during gestation (Hidiroglou and Knipfel, 1981), and neonates depend on liver stores of trace minerals for postnatal utilization due to reduced concentrations of these elements in colostrum and milk (Underwood and Suttle, 1999). Indeed, liver concentrations of Co, Cu, Mn, or Zn decreased in calves from AAC and INR cows when comparing samples from birth and 24 h later (day effect, *P* < 0.01). Collectively, these outcomes suggest similar transfer of Co, Cu, Mn, or Zn from maternal to fetal tissues during gestation, and supply of these trace minerals for colostrogenesis between AAC and INR cows (Hostetler et al., 2003). Marques et al. (2016a) also indicated equivalent transfer of Co, Cu, Zn, and Mn from maternal to fetal tissues between cows receiving AAC and INR during late gestation, based on similar concentrations of these trace minerals in cotyledons and in the newborn liver. Therefore, supplementing organic-complexed Co, Cu, Zn, and Mn appears to yield a similar supply of these trace elements to fetal tissues and the mammary gland compared with inorganic sulfate sources.

**Table 9.** Concentrations of Co, Cu, Mn, and Zn in cotyledons and liver from newborn calves born from beef cows that received diets containing supplemental INR (*n* = 95) or AAC (*n* = 95) during gestation^1,2^

Item	INR	AAC	SEM	*P-*value
Co, mg/kg				
Cotyledon	0.494	0.533	0.090	0.77
Calf birth	0.202	0.188	0.015	0.51
Calf 24 h after birth	0.153	0.148	0.010	0.73
Calf early life	0.223	0.228	0.010	0.73
Calf weaning	0.166	0.165	0.009	0.94
Cu, mg/kg				
Cotyledon	8.38	9.07	0.92	0.61
Calf birth	394	399	19	0.87
Calf 24 h after birth	303	291	20	0.67
Calf early life	84.5	75.9	14.3	0.67
Calf weaning	70.4	73.7	9.3	0.80
Mn, mg/kg				
Cotyledon	18.9	18.4	5.46	0.95
Calf birth	5.96	6.06	0.34	0.83
Calf 24 h after birth	4.70	4.83	0.29	0.75
Calf early life	11.6	12.0	0.5	0.44
Calf weaning	9.19	8.80	0.19	0.17
Zn, mg/kg				
Cotyledon	90.9	93.4	3.3	0.60
Calf birth	823	869	79	0.69
Calf 24 h after birth	676	637	79	0.73
Calf early life	154	148	9	0.59
Calf weaning	137	142	5	0.42

^1^INR and AAC cows received the same amount of supplemental Co, Cu, Mn, and Zn from sulfate sources or Availa 4 (Zinpro Corporation, Eden Prairie, MN). Cows were assigned to the experiment at 117 ± 2 d of gestation (day 0).

^2^Cotyledon and calf liver samples (needle biopsy; Arthington and Corah, 1995) were collected within 8 h after calving. A total of 32 placentas were retrieved (16 placentas/treatment) for cotyledon collection. Calf liver samples were collected again 24 h after birth, when calves were 43 ± 0.5 d of age (early life), and at weaning (day 367). Concentrations of Co, Cu, Mn, and Zn were determined by the Michigan State University Diagnostic Center for Population and Animal Health (East Lansing, MI; Braselton et al., 1997). All liver samples were collected from 30 cows of each treatment randomly selected on each sampling day.

No treatment effects were detected (*P* ≥ 0.38) for mRNA expression of hepatic *CUT*, *MT*, or *SOD* in calves at birth or 24 h later (Table 10), which suggest similar hepatic Cu and Zn metabolism during early life (Coyle et al., 2002; Han et al., 2009; Hansen et al., 2009). Day effects were also observed (*P* < 0.01) for calf liver mRNA expression of *CUT* and *MT*, both of which increased 24 h after birth across treatments reflecting the greater activity in hepatic tissue and utilization of trace minerals in the neonate (Underwood and Suttle, 1999; López-Alonso et al., 2005). No treatment differences were detected (*P* ≥ 0.29) for mRNA expression of LM genes associated with adipogenic or muscle development activities at birth (Table 11). *Myogenin* is a regulatory factor that influences postnatal muscle growth through differentiation and fusion of satellite cells with existing fibers (Le Grand and Rudnicki, 2007; Du et al., 2010). These cells, both quiescent and activated, are marked by the expression of *paired box gene 7* (***PAX7***), which is necessary for satellite cell specification and survival (Seale et al., 2000; Li et al., 2011). Trace minerals, such as Zn, are required for muscle differentiation through the activation and proliferation of satellite cells (Petrie et al., 1996; Ohashi et al., 2015). *Peroxisome proliferator-activated receptor gamma* (***PPAR-γ***) plays a pivotal role in adipocyte differentiation and associated gene expression (Houseknecht et al., 2002), the process of which is initiated around mid-gestation in the ruminant (Du et al., 2010). *Adipocyte fatty acid-binding protein* (***FABP4***) is a target of PPAR-γ and highly involved in the differentiation of adipocytes by acting as an intracellular fatty acid chaperone (Michal et al., 2006). Several Zn-finger proteins participate in adipocyte determination and differentiation (Wei et al., 2013), while Zn has been shown to enhance adipogenesis in vitro (Tanaka et al., 2001). Despite the established role of trace minerals on muscle development or adipogenesis, supplementing gestating cows with Cu, Co, Mn, or Zn as organic-complexed instead of sulfate sources did not alter the mRNA expression of genes associated with these activities in the LM of neonatal calves.

**Table 10.** Expression of liver genes in calves born from beef cows that received diets containing supplemental INR (*n* = 95) or AAC (*n* = 95) during gestation^1,2^

Item	INR	AAC	SEM	*P-*value
*CUT*				
Birth	2.19	2.23	0.11	0.78
24 h after birth	2.51	2.53	0.11	0.91
Weaning	2.13	2.01	0.10	0.40
*MT*				
Birth	33.7	32.9	7.2	0.94
24 h after birth	59.4	68.4	7.2	0.38
Weaning	39.9	54.1	8.8	0.26
*SOD*				
Birth	2.92	2.96	0.20	0.88
24 h after birth	2.77	2.70	0.20	0.79
Weaning	1.78	1.98	0.07	0.04

^1^INR and AAC cows received the same amount of supplemental Co, Cu, Mn, and Zn from sulfate sources or Availa 4 (Zinpro Corporation, Eden Prairie, MN). Cows were assigned to the experiment at 117 ± 2 d of gestation (day 0).

^2^Liver samples were collected via biopsy (Arthington and Corah, 1995) at birth, 24 h after birth, and at weaning (day 367). Values are expressed as relative fold change compared within CT of reference genes analyzed within the same sample (Ocón-Grove et al., 2008). Samples were collected from 30 cows of each treatment randomly selected on each sampling day.

**Table 11.** Expression of LM genes in calves born from beef cows that received diets containing supplemental INR (*n* = 95) or AAC (*n* = 95) during gestation^1,2^

Item	INR	AAC	SEM	*P-*value
*FABP4*				
Birth	155.7	108.0	31.4	0.29
Weaning	45.9	38.4	9.0	0.56
*Myogenin*				
Birth	5.05	4.54	0.58	0.53
Weaning	4.20	4.42	0.51	0.77
*PAX7*				
Birth	3.42	3.42	0.25	0.99
Weaning	3.23	3.22	0.16	0.98
*PPAR-γ*				
Birth	4.28	4.24	0.76	0.97
Weaning	2.14	2.03	0.21	0.71

^1^INR and AAC cows received the same amount of supplemental Co, Cu, Mn, and Zn from sulfate sources or Availa 4 (Zinpro Corporation, Eden Prairie, MN). Cows were assigned to the experiment at 117 ± 2 d of gestation (day 0).

^2^Muscle samples were collected via needle biopsy (Schubach et al., 2019) at birth and at weaning (day 367). Values are expressed as relative fold change compared within CT of reference genes analyzed within the same sample (Ocón-Grove et al., 2008). Samples were collected from 30 cows of each treatment randomly selected on each sampling day.

No treatment differences were detected (*P* ≥ 0.44) for liver concentrations of Co, Cu, Mn, and Zn in calves the day after WSW (early life; Table 9). These results agree with the hepatic trace mineral profile of newborn calves, trace mineral profile of maternal milk at WSW, and similar nutritional management that cow–calf pairs received after calving. At weaning, no treatment differences were detected (*P* ≥ 0.18) for weaning rate, proportion of AI-sired and male calves weaned, weaning age, and calf weaning BW (Table 8). Liver concentrations of Co, Cu, Mn, and Zn also did not differ (*P* ≥ 0.17) between treatments (Table 9). Hepatic mRNA expression of SOD at weaning was greater (*P* = 0.04) in calves from AAC cows compared with INR cohorts, while no treatment differences (*P* ≥ 0.26) were detected in the hepatic mRNA expression of *CUT* and *MT* (Table 10) nor mRNA expression of *FABP4*, *myogenin*, *PAX7*, and *PPAR-γ i*n the LM (Table 11). These outcomes suggest that supplementing AAC instead of INR to gestating beef cows did not yield immediate and longer-term effects in hepatic trace mineral profile and LM expression of adipogenic and muscle development genes, resulting in similar calf growth until weaning. The upregulated hepatic mRNA expression of *SOD* in calves from AAC may have impacted postweaning responses, which are discussed in the companion manuscript (Harvey et al., 2021).

Marques et al. (2016a) also did not report differences in weaning responses between cows supplemented with INR or AAC during late gestation, despite a 13-kg numerical increase in weaning BW from AAC cows. This experiment was designed with a greater number of cows and providing AAC and INR during a longer period during gestation to expand on the results reported by Marques et al. (2016a). One can speculate that cow BCS loss during gestation observed herein may have limited the benefits of the AAC treatment, given the importance of cow nutritional status to postnatal offspring development (Bohnert et al., 2013; Marques et al., 2016b). As an attempt to address this question, AAC and INR cows were divided according to BCS change during gestation (day −30 to 97). Calf weaning BW also did not differ (*P* ≥ 0.51) between AAC and INR cows (*n* = 129) that lost a significant amount of BCS (−1.67 and −1.74 BCS change, SEM = 0.07; 182 vs. 183 kg of BW, SEM = 3; respectively) or cows (*n* = 43) whose BCS change was below the sensitivity of the BCS scale (−0.42 and −0.48 BCS change, SEM = 0.08; 176 vs. 178 kg of BW, SEM = 6; respectively). Therefore, the lack of differences in offspring productivity between AAC and INR cows is likely not associated with the unexpected cow BCS loss during gestation observed in this experiment.

### Overall conclusions

Supplementing beef cows with AAC during gestation did not improve liver concentrations of these trace minerals nor enhanced the transport of these trace minerals to the fetus or colostrum compared with cohorts supplemented with sulfate sources. No major physiological and productive benefits were realized from cows receiving organic-complexed trace minerals, besides upregulated mRNA expression of hepatic genes associated with Cu and Zn metabolism at calving and in their offspring at weaning. The results from this manuscript indicate that supplementing Co, Cu, Zn, and Mn as organic-complexed or sulfate sources to beef cows during the last 5 mo of gestation yielded similar cow–calf productive responses until weaning. The companion manuscript (Harvey et al., 2021) describes the postweaning responses of the female and male offspring reared, respectively, as replacement heifers and feeder cattle.

## Abbreviations

AAC: organic-complexed source of Cu, Co, Mn, and Zn
AI: artificial insemination
BCS: body condition score
BW: body weight
INR: sulfate sources of Cu, Co, Mn, and Zn
LM: longissimus muscle
PCR: polymerase chain reaction
WSW: weigh–suckle–weigh
